# Association between penicillin allergy labels and serious adverse events in hospitalized patients: a systematic review and meta-analysis

**DOI:** 10.3389/fphar.2024.1519522

**Published:** 2025-01-06

**Authors:** Shipeng Zhang, Tianyi Dong, Jiawen Xian, Xinyue Xiao, Jiaqing Yuan, Tong Zeng, Kuan Deng, Rui Fu, Hanyu Wang, Yanjie Jiang, Xueying Li

**Affiliations:** ^1^ Clinical Medical College, Chengdu University of Traditional Chinese Medicine, Chengdu, China; ^2^ Hospital of Chengdu University of Traditional Chinese Medicine, Chengdu University of Traditional Chinese Medicine, Chengdu, China; ^3^ Nanjing Hospital of Chinese Medicine Affiliated to Nanjing University of Chinese Medicine, Nanjing, China

**Keywords:** penicillin allergy labels, adverse events, meta, relative risk, mortality

## Abstract

**Background:**

To date, several studies have demonstrated that erroneous labeling of Penicillin allergy (PAL) can significantly impact treatment options and result in adverse clinical outcomes, while other studies have reported no negative effects. Therefore, to systematically evaluate these effects and investigate the association between adverse clinical outcomes and the Penicillin label, we conducted this meta-analysis.

**Method:**

Searches were conducted in the PubMed, Embase, Cochrane Library, and Web of Science databases from inception to 13 July 2024. The search strategy utilized terms (“antibiotic allergy label,” “penicillin allergy label,” and “allergy label”) and (“death,” “readmission,” “adverse outcome,” and “clinical adverse outcome”). In the study selection process, the PICOS framework and stringent inclusion/exclusion criteria were applied. The quality of the initially included studies was independently assessed using the Newcastle-Ottawa Scale (NOS). Data from the included studies, including relative risk (RR) and 95% confidence intervals (CI), were extracted and analyzed using Stata 16.0. Sensitivity analyses were conducted to validate the results. Heterogeneity was assessed using the I^2^ and Cochrane Q tests, and publication bias was evaluated using Egger’s test and funnel plot analysis.

**Results:**

A total of 497 relevant studies were identified through four databases. Following a thorough screening process, 11 studies encompassing 1,200,785 participants were ultimately included. The combined evidence suggests that penicillin allergy labeling is associated with increased mortality RR = 1.06 (95% CI 1.06–1.07, I^2^ = 0.00%), acute heart failure (RR = 1.19, 95% CI 1.09–1.30, τ^2^ = 0.00, I^2^ = 92.39%), ICU events (RR = 1.10, 95% CI 1.01–1.19, τ^2^ = 0.00, I^2^ = 57.09%), and mechanical ventilation events (RR = 1.16, 95% CI 1.09–1.24, τ^2^ = 0.00, I^2^ = 23.11%). Additionally, there was no significant association with readmissions (RR = 1.05, 95% CI 0.95–1.16, I^2^ = 0.00%).

**Conclusion:**

Our findings indicate that penicillin allergy labels are associated with an increased risk of mortality in patients, as well as being linked to acute heart failure, heightened ICU requirements, and mechanical ventilation.

**Systematic Review Registration:**

PROSPERO, identifier CRD42024571535. Available from: https://www.crd.york.ac.uk/prospero/display_record.php?ID=CRD4202457153.

## Introduction

Since the discovery of antibiotics nearly a century ago, researchers have identified during extensive clinical use that adverse drug reactions (ADR) and hypersensitivity reactions (HSR) associated with antibiotics are key factors limiting their clinical application (Blumenthal et al., 2019; Lucas et al., 2024). Mild skin reactions (such as rashes, itching) to severe systemic reactions (such as anaphylactic shock or difficulty breathing) are typically considered signs of antibiotic allergy and are recorded in electronic health records or retained by the patient. It is important to note that common drug side effects (such as nausea, vomiting, and fever) are sometimes inaccurately recorded as allergies, potentially leading to the use of unnecessarily broad-spectrum or suboptimal antibiotics, which pose a significant risk to patient safety and increase the public health burden (Blumenthal et al., 2019).

Currently, the most widely studied and longest used antibiotics are beta-lactam antibiotics, which are also the most common culprits of HSR (Bigby et al., 1986). However, antibiotic HSR is frequently misdiagnosed due to the presence of rashes, which can also result from viral infections (e.g., herpesviridae), bacterial infections (e.g., *Streptococcus* pyogenes), or drug-infection interactions (Chovel-Sella et al., 2013; Thompson and Ramos, 2017; Hocqueloux et al., 2013).

Penicillin, a member of the beta-lactam class of antibiotics, is the most commonly associated with allergic reactions. A de-labeling study of penicillin allergy label (PAL) found that 97.13% of 1,070 patients were successfully de-labeled (Stul et al., 2024). A large number of erroneous PALs profoundly affect treatment options, as many patients are incorrectly labeled as allergic to penicillin, limiting healthcare workers’ antibiotic choices and leading to the use of alternative drugs. In a population-matched cohort study, Kimberly G. Blumenthal found that PAL patients had higher usage rates of alternative antibiotics: macrolides at 4.15 (95% CI 4.12–4.17), clindamycin at 3.89 (95% CI 3.66–4.12), and fluoroquinolones at 2.10 (95% CI 2.08–2.13). These patients were also more likely to develop methicillin-resistant *Staphylococcus aureus* and *Clostridium difficile* infections (Blumenthal et al., 2018). Moreover, these alternative treatments often result in longer hospital stays and a heightened risk of serious adverse reactions (van Dijk et al., 2016; Kirven et al., 2021).

To date, no systematic review has comprehensively evaluated the evidence. Some studies indicate an association between PAL and increased mortality (Kirven et al., 2021), while others find no such link, resulting in conflicting outcomes that pose significant challenges for clinical guidance (Beddow et al., 2022; Conway et al., 2017). Therefore, we analyzed the clinical adverse events associated with penicillin allergy labeling to offer valuable guidance for the clinical use of penicillin and the rationalization of PAL.

## Methods and materials

### Registration of review protocol

This study adhered to the Preferred Reporting Items for Systematic Reviews and Meta-Analyses (PRISMA) 2020 guidelines (Page et al., 2021), which is a systematic review and meta-analysis of observational studies investigating the association between penicillin allergy labeling and adverse outcomes. The research protocol has been registered with PROSPERO, the international registry for systematic reviews (Registration No. CRD 42024571535).

### Search strategy

Searches were conducted in the PubMed, Embase, Cochrane Library, and Web of Science databases from inception to 13 July 2024. The search was restricted to publications in English. The search strategy utilized Medical Subject Headings (MeSH) and free-text combinations related to exposure (“antibiotic allergy label,” “penicillin allergy label,” and “allergy label”) and outcomes (“death,” “readmission,” “adverse outcome,” and “clinical adverse outcome”). References from articles on similar topics were manually reviewed to identify additional qualifying studies. Detailed search strategies are provided in Appendix 1.

### Eligibility criteria

Following the recommendation (Dekkers et al., 2019), the PECO(S) framework was applied to define the review question (Morgan et al., 2018). The study included any population, with no restrictions on age (>18 years), sex (male or female), or pregnancy status (P). Participants were labeled with the penicillin allergy label (E), and the probability of adverse clinical events was compared to that of a group without allergy labels (C). In-hospital mortality, readmission, ICU admission, mechanical ventilation, and risk of acute heart failure were the outcomes of interest (O). We focused exclusively on reports from observational studies (S).

The following exclusion criteria were applied: 1) duplicate literature or studies reporting the same cohort; 2) studies without full-text or available data; 3) studies focusing on populations with relevant diseases; 4) unrelated exposures, such as other antibiotic exposure (e.g., sulfonamides, vancomycin); 5) outcomes not related to the exposure (e.g., frequency of broad-spectrum antibiotic use); and 6) non-relevant study designs (e.g., intervention studies, reviews, meta-analyses, study protocols, letters, or case reports).

### Study selection

The literature screening process was divided into two steps. First, two authors (ZSP and DTY) conducted a comprehensive search of the relevant literature. All identified literature was imported into EndNote X9, and duplicates were removed both automatically and manually. Eligible studies were identified by screening titles and abstracts based on predefined inclusion and exclusion criteria. The second step involved reviewing studies of uncertain eligibility through full-text screening to determine their suitability for inclusion in the meta-analysis. In cases of disagreement during the selection process, the third author (XJW) was consulted until consensus was reached.

### Data extraction and quality assessment

The first author employed standardized data collection tables to extract relevant data from eligible studies, whereas the second author conducted an independent verification of this data against the original articles. Specifically, the extracted information includes the following: the first author’s last name and year of publication, the study name (if applicable), geographical location, study time interval, specific study design, demographic characteristics, sample size, exposure outcomes (in-hospital mortality, readmission, ICU admission, mechanical ventilation, acute heart failure), fully adjusted risk ratios, adjusted confounders in statistical models, and risk diseases among confounders. The final data extraction process was based on the consensus between ZSP and DTY. The quality of the initially included studies was assessed independently using the Newcastle-Ottawa Scale (NOS). The NOS comprises eight items categorized into three dimensions (selection, comparability, and outcome), and the criteria assign a maximum of 9 points for each study, allocated as 4 points for selection, 2 points for comparability, and 3 points for outcome evaluation. Studies receiving scores of 0–3, 4–6, and 7–9 are classified as low quality, medium quality, and high quality, respectively. Additionally, according to the Grading of Recommendations, Assessment, Development and Evaluation (GRADE), we evaluated evidence of each health outcome and graded it as “high,” “moderate,” “low,” or “very low” quality to draw conclusions.

In this meta-analysis, we conducted a detailed and rigorous screening of duplicate data sources and exposure indicators to ensure the independence and reliability of the 12 included studies. For 3 studies with potential overlap in data sources, we applied the following rigorous treatment strategy, and finally 3 studies were included in the main meta-analysis: (1) If both studies were from the same cohort, they were considered separate studies if they included different populations (age). (2) If two studies are from the same cohort, they are considered independent studies if the included population is exposed to different diseases.

### Data synthesis and analysis

We employed the DerSimonian-Laird random effects model (DerSimonian and Laird, 1986). The relative risk (RR) and 95% confidence interval (CI) for penicillin allergy labeling and clinical adverse event risk were summarized to account for variability across studies. When heterogeneity was 0, the inverse variance fixed-effects model was selected to compare differences between the fixed-effects and random-effects models and assess the appropriateness of the analysis method. A two-tailed *p*-value < 0.05 was considered statistically significant. We used the I^2^ and Cochrane Q tests to quantify heterogeneity. To assess the significance of RR differences and the potential impact of residual confounders, a “leave-one-out” sensitivity analysis was performed by omitting one study per iteration to evaluate the influence of individual studies on the overall effect. Egger’s test (Egger et al., 1997) and a funnel plot analysis were used to detect publication bias. We considered a *p*-value < 0.05 or asymmetry in the funnel plot as indicative of potential publication bias. If publication bias was detected, the trim-and-fill method was applied to assess its impact on the reliability of the results.

### Software, data, and code availability

We used Stata 16.0 (Stata Corp, College Station, Texas) for the meta-analysis.

## Result

**FIGURE 1:**
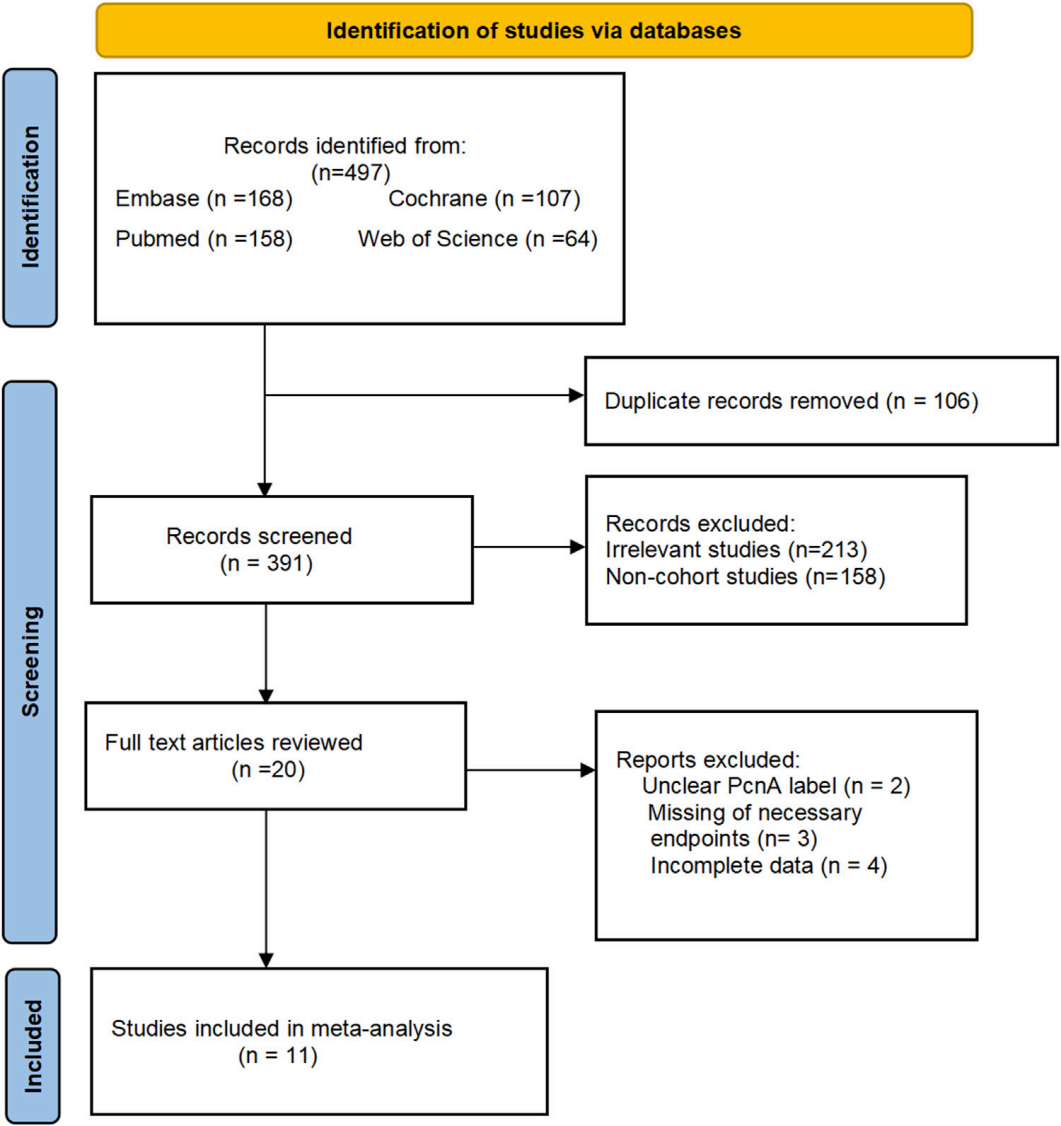
Flow chart.

A preliminary search conducted across four databases identified a total of 497 relevant studies. Following the removal of 106 duplicates and the exclusion of 380 studies through full-text screening, a total of 11 relevant studies were ultimately included, encompassing 1,200,785 participants (Figure 1) (van Dijk et al., 2016; Kirven et al., 2021; Beddow et al., 2022; Conway et al., 2017; Baman et al., 2012; Kaminsky et al., 2023; Kaminsky et al., 2022; Motoa et al., 2019; Nelson et al., 2022; Pérez-Encinas et al., 2022; Sousa-Pinto et al., 2018). A meta-analysis was conducted on the five primary outcomes, with publication bias and sensitivity analyses specifically undertaken for in-hospital mortality, the primary outcome. Given the limited number of included studies, other relevant analyses were considered less significant. Basic information of the 11 studies included in the analysis is shown in Table 1. The quality of the 11 included studies was assessed, revealing one study rated as high quality and ten rated as medium quality (Table 2).

**TABLE 1:** Basic information of literature.

Author. year	Date from	Country	Total, n	Population	Outcome	Population complications	Effect size
Savannah M van Dijk 2016	Utrecht Patient Orientated Database	Netherlands	3,936	Adult	Mortality, Readmission	Hospitalized non-critical patients	RR for mortality 1.49 (0.58,3.85), readmission 1.19 (0.98,1.44)
David Beddow 2022	11-hospital system in Minnesota and Western Wisconsin	Canada	5,238	Adult	Mortality, Mechanical ventilation, ICU, Readmission	Sepsis	RR for mortality 1.04 (0.73,1.47), mechanical ventilation 0.95 (0.73,1.23), ICU 0.97 (0.86,1.09), readmission 1.03 (0.9,1.17)
Erin L Conway 2017	ED and admitted to the Veterans Affairs Western New York Healthcare System	America	403	Elder people	Readmission	Pneumonia, urinary tract infection, bacteremia, and sepsis	RR for readmission 0.99 (0.52, 1.91)
Justin Kirven 2021	The Allina Health system	America	4,387	Adult	ICU, Readmission	Group B *streptococcus*	RR for ICU 0.85 (0.05, 15.79), readmission 0.76 (0.48, 1.19)
Baman 2012	Data of inpatient hospital cases	America	2,550	Elder people	Mortality	Pneumonia	RR for mortality 1.47 (0.91, 2.39)
Lauren W Kaminsky 2021	TriNetX (Patients from 48 healthcare organizations across the United States)	America	13,168	Adult	Acute respiratory failure, Mechanical ventilation, Mortality, ICU	COVID-19	RR for acute respiratory failure 1.25 (1.19, 1.31), mechanical ventilation 1.17 (1.03, 1.32), mortality 1.09 (0.96, 1.23), ICU 1.2 (1.08, 1.34)
Jared Nelson 2022	National Inpatient Sample database	America	50,069	Adult	Mortality	Kidney transplant	RR for mortality 0.61 (0.39, 0.95)
Montserrat Pérez-Encinas 2022	Spanish Hospital System	Spain	981,291	Adult	Mortality	At least one infectious disease	RR for mortality 0.834 (0.825, 0.844)
Bernardo Sousa-Pinto 2018	Database containing all hospitalizations in Portuguese public hospitals	Porto	3,438	<18 years	Mortality	Hospitalization associated infection	RR for mortality 2.94 (0.31, 28.19)
Motoa 2019	Data of inpatient hospital cases	America	1,809	Adult	Readmission	liver transplant recipients	RR for readmission 0.95 (0.64, 1.16)
Lauren W. Kaminsky 2023	TriNetX (Patients from 48 healthcare organizations across the United States)	America	137,496	Adult	Acute respiratory failure, Mechanical ventilation, Mortality, ICU	Bacterial Pneumonia	RR for acute respiratory failure 1.14 (1.12, 1.15), ICU 1.11 (1.08, 1.14), mortality 1.08 (1.04, 1.13), mechanical ventilation 1.18 (1.13, 1.22)

**TABLE 2:** Newcastle—ottawa quality assessment scale (cohort studies).

Author year	Selection				Comparability	Outcome			Total score	Overall quality
	1	2	3	4	1	1	2	3		
Savannah M van Dijk 2016	1	1	1	1	0	1	0	0	5	★★
David Beddow 2022	1	1	1	1	1	1	0	0	6	★★
Erin L Conway 2017	0	1	1	1	1	1	0	0	5	★★
Justin Kirven 2021	1	1	0	1	1	1	0	0	5	★★
Baman 2012	1	1	1	1	0	1	0	0	5	★★
Lauren W Kaminsky 2021	1	1	1	1	2	1	0	0	7	★★★
Jared Nelson 2022	1	1	1	1	0	1	0	0	5	★★
Montserrat Pérez-Encinas 2022	1	1	1	1	1	1	0	0	6	★★
Bernardo Sousa-Pinto 2018	1	1	1	1	1	1	0	0	6	★★
Motoa 2019	0	0	1	1	1	1	0	0	4	★★
Lauren W. Kaminsky 2023	1	1	1	1	1	1	0	0	6	★★

Wells GA, Shea D, O'Connell D, et al. The newcastle-ottawa scale (NOS) for assessing the quality of nonrandomised studies in meta-analyses. https://www.ohri.ca/programs/clinical_epidemiology/oxford.asp.

Selection: 1: Representativeness of the exposed cohort (1 point); 2: Selection of the non exposed cohort (1 point); 3: Ascertainment of exposure (1 point); 4: Demonstration that outcome of interest was not present at start of study (1 point).

Comparability: 1: Comparability of cohorts on the basis of the design or analysis (2 point).

Outcome: 1: Assessment of outcome (1 point); 2: Was follow-up long enough for outcomes to occur (1 point); 3: Adequacy of follow up of cohorts (1 point).

★ = Low quality (0–3 point), ★★ = Moderate quality (4–6 point), ★★★ = High quality (7–9 point).

**FIGURE 2:**
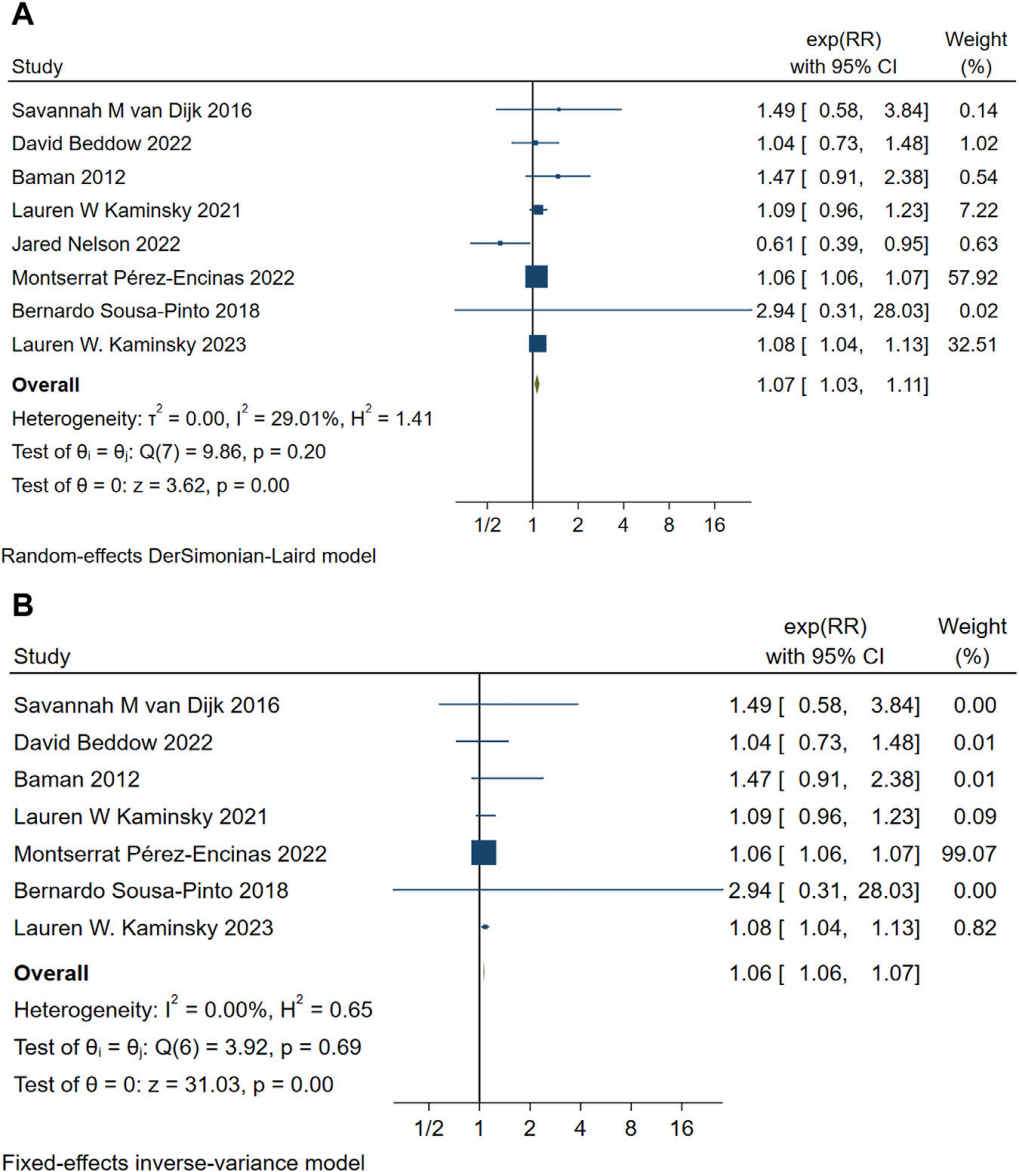
**(A)** Forest map of PAL and mortality. **(B)** Summary forest maps of PAL and mortality after excluding one included article.

Eight studies analyzed the relationship between PAL and mortality (van Dijk et al., 2016; Beddow et al., 2022; Baman et al., 2012; Kaminsky et al., 2023; Motoa et al., 2019; Nelson et al., 2022; Sousa-Pinto et al., 2018; Kaminsky et al., 2021). The pooled analysis demonstrated an association between penicillin allergy labeling and mortality (RR = 1.07, 95% CI 1.03–1.11, τ^2^ = 0.00, I^2^ = 29.01%) as shown in Figure 2A. The sensitivity analysis indicated that the results were not robust, The sensitivity analysis indicated that the results were not robust, the Jared Nelson 2022 study was a possible source of the unreliable results. After excluding studies involving cooperative severe surgeries [e.g., kidney transplantation (Nelson et al., 2022)] as shown in Figure 2B, the pooled estimate was RR = 1.06 (95% CI 1.06–1.07, I^2^ = 0.00%), with significantly reduced heterogeneity. Sensitivity analysis revealed no conflicting outcomes after the exclusion of any study, indicating robust results. The combined evidence suggests that penicillin allergy labeling is associated with increased mortality, with the difference being statistically significant.

**FIGURE 3:**
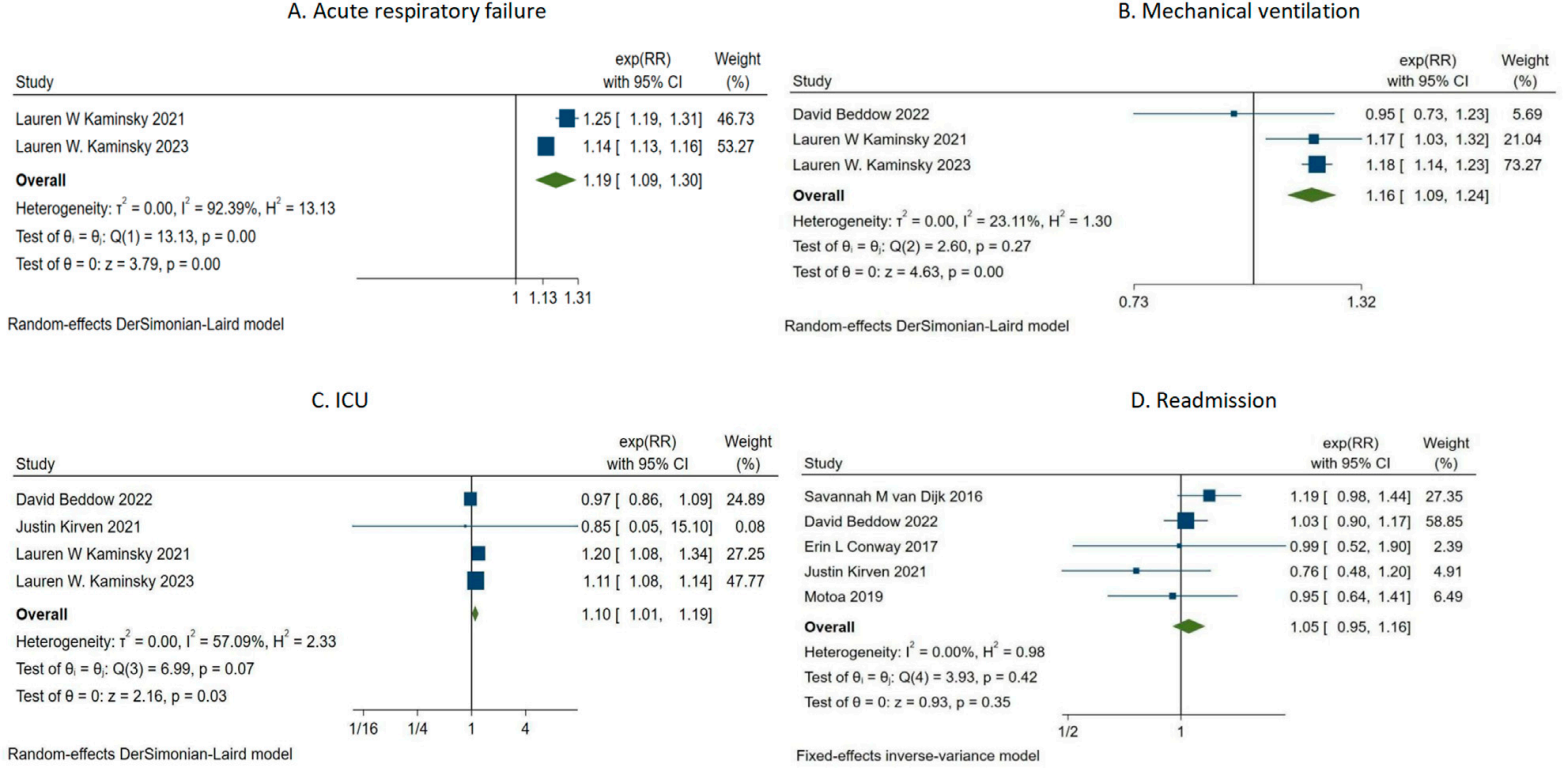
Forest map of PAL and Four serious adverse clinical events.

The summary analysis of PAL and four clinical adverse events is shown in Figure 3. Two studies (Kaminsky et al., 2023; Kaminsky et al., 2022) investigated the relationship between PAL and acute heart failure (Figure 3A). The summary analysis indicated a significant association between PAL and acute heart failure (RR = 1.19, 95% CI 1.09–1.30, τ^2^ = 0.00, I^2^ = 92.39%). Three studies (Beddow et al., 2022; Kaminsky et al., 2022; Kaminsky et al., 2021) assessed the correlation between penicillin allergy labeling and mechanical ventilation events (Figure 3B). The summary analysis revealed a correlation between penicillin allergy labeling and mechanical ventilation events (RR = 1.16, 95% CI 1.09–1.24, τ^2^ = 0.00, I^2^ = 23.11%). Four studies (Kirven et al., 2021; Beddow et al., 2022; Kaminsky et al., 2023; Kaminsky et al., 2022) evaluated the association between penicillin allergy labeling and ICU events (Figure 3C). The pooled analysis demonstrated an association between PAL and ICU events (RR = 1.10, 95% CI 1.01–1.19, τ^2^ = 0.00, I^2^ = 57.09%). Five studies (van Dijk et al., 2016; Kirven et al., 2021; Beddow et al., 2022; Conway et al., 2017; Motoa et al., 2019) investigated the association between penicillin allergy labeling and readmissions (Figure 3D). The summary analysis found no significant association between penicillin allergy labeling and readmissions (RR = 1.05, 95% CI 0.95–1.16, I^2^ = 0.00%).

### Publication bias

**FIGURE 4:**
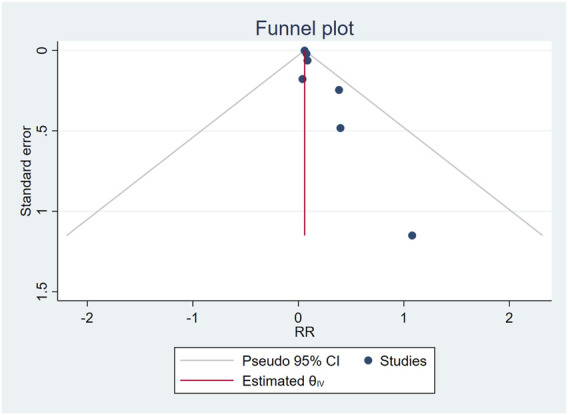
Publication bias.

**FIGURE 5:**
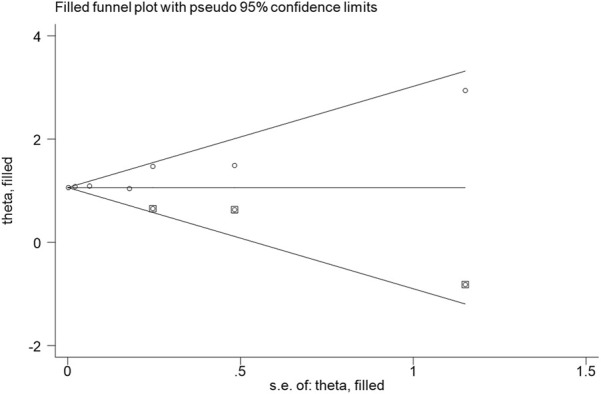
Shear compensation.

The combination of funnel diagram (Figure 4) and egger test analysis results (*p* = 0.019 < 0.05) suggested that there was publication bias in the included literature, and the clip-supplement method was used for verification (Figure 5). The results did not change, indicating that the results of the article were robust.

### GRADE evaluation

According to the GRADE scoring system, observational studies were rated beginning at Grade C, with final results indicating that the evidence for penicillin allergy labeling and mortality was rated as Grade C, while all remaining outcomes were rated as Grade D (indicating inconsistencies, inaccuracies, and partial heterogeneity in the study results, leading to a reduced score).

## Discussion

This was the first meta-analysis of PAL and clinical adverse events, assessing five adverse events including readmission, ICU admission, mechanical ventilation, acute heart failure, and death through a pooled review of 1^2^ included articles.

Our findings indicated that PAL was associated with an increased risk of mortality in patients, a result that was concerning. PAL was associated with a higher risk of mortality (RR = 1.06, 95% CI: 1.06–1.07); However, any risk factor that contributed to mortality remained a significant concern for clinicians and patients. In comparison to previous studies, two distinct population studies utilizing the TriNetX data cohort found a significant association (Kaminsky et al., 2022; Kaminsky et al., 2021), while another study that included multiple antibiotic allergy labels also identified a relationship between allergy labels and mortality events (Charneski et al., 2011). Other studies suggested a potential association between PAL and increased mortality; However, they did not demonstrate a statistically significant difference (van Dijk et al., 2016; Beddow et al., 2022; Baman et al., 2012). Similarly, the PAL may also have been linked to adverse events, including acute heart failure, increased ICU requirements, and the need for mechanical ventilation. Generally, these three types of events were interrelated; when acute heart failure occurred, patients often required admission to the ICU and mechanical ventilation to sustain life.

The allergy label itself suggested that the patient’s immune system had an abnormal or overreactive tendency (Rose et al., 2024). These immune abnormalities were not limited to drug allergies but also manifested as sensitivities to other external factors that increased the risk of systemic inflammation (Blumenthal et al., 2019). Chronic inflammation was an important cause of cardiovascular disease, including heart failure. On the other hand, when a patient was labeled allergic to penicillin, doctors had to choose alternative antibiotics, such as cephalosporins, vancomycin, or quinolones. These alternative drugs were sometimes more likely to cause adverse reactions than penicillin, especially in patients with underlying cardiovascular disease. These drugs could cause direct or indirect stress on the heart, for example, by increasing the burden on the heart, causing electrolyte disturbances, or triggering an inflammatory response that increased the risk of heart failure.

This analysis indicated that the PAL was associated with the majority of serious clinical adverse events, and previous studies had shown that PAL was also associated with longer hospital stays and increased drug-resistant bacterial infections. The emergence of various adverse reactions suggested that the de-labeling of penicillin allergy labels should be further promoted. Studies had shown that the actual prevalence of penicillin allergy among individuals labeled as penicillin allergic was only about 10%, and more than 90% of patients labeled with penicillin allergy were found to be non-allergic after assessment by a qualified allergist (Ghiordanescu et al., 2024; Drummond et al., 2024). Similarly, a penicillin allergy trial conducted at the University of Montpellier indicated that out of 1,884 participants with a history of penicillin allergy, only 382 (20.3%) showed a positive penicillin allergy test (Ghiordanescu et al., 2024). Misdiagnosis of allergy labels was a widespread trend that caused significant distress to clinical protocols and even increased the risk of adverse events. In particular, patients who were not truly allergic but were simply labeled as allergic due to adverse reactions or family history should have undergone skin testing or allergy testing to reduce the waste of medical resources caused by mislabeling and unnecessary alternative treatments (Arasaratnam et al., 2024). In addition, the results of this analysis would also play a role in promoting the implementation of allergy labels.

### Penicillin allergy labels with other clinical events

#### Impact on the outpatient clinics

PAL is associated with higher rates of prescribing broad-spectrum and second-line antibiotics to children treated for respiratory infections in primary care, potentially increasing the risk of treatment failure (Joerger et al., 2023). Remarkably, children were assigned labels early in life, with nearly half receiving this designation (Taylor et al., 2022) after having received one or no penicillin prescriptions. These findings raise important questions regarding the effectiveness of penicillin allergy labels. One study surprisingly found that primary care physicians and patients frequently suspected that allergy records were inaccurate, yet physicians were often reluctant to amend these records (Wanat et al., 2021).

#### The use of broad-spectrum antibiotics and the increased risk of resistant bacteria

After adjusting for age and diagnosis, the odds of receiving a second-line antibiotic were 137 (95% CI 112–169) higher for patients with penicillin allergy compared to those without in Adam L Hersh’s research (Hersh et al., 2020). Several regional studies have also indicated that patients labeled with PAL are more likely to receive broad-spectrum antibiotics (Jiang et al., 2022; Trubiano et al., 2016). Furthermore, the overuse of broad-spectrum antimicrobials can increase the risk of infections, such as methicillin-resistant *S. aureus* (MRSA) and *C. difficile* (Blumenthal et al., 2018; Trubiano et al., 2016). Professor Thomas Hills conducted a follow-up study with an average duration of 4.55 years, during which 215 out of 304 (70.7%) of the de-labeled patients received penicillin antibiotics. The proportion of antibiotic courses involving penicillin increased from 12.81% to 39.62% in this group. Following de-labeling, the incidence of macrolides, cephalosporins, trimethoprim/trimethoprim-sulfamethoxazole, fluoroquinolones, “other” non-penicillin antibiotics, and broad-spectrum antibiotic use were all reduced (Hills et al., 2020).

#### Choice of antibiotics during surgery and surgical infection

The study demonstrated that, compared to non-PAL surgeries, clindamycin and vancomycin were used more frequently (Coleman et al., 2020; Yian et al., 2020). Multiple studies on perioperative clindamycin use in patients with allergy labels have found that PAL is associated with an increased risk of surgical site infection (Yian et al., 2020; Seidelman et al., 2022; Roebke et al., 2022). It is important to note that, in the context of gastrointestinal surgery, studies have yielded differing outcomes. Two studies on PAL and gastrointestinal surgery found no correlation between PAL and surgical site infection (Khan et al., 2022; Jones et al., 2024). The difference in outcomes may be due to the choice of antibiotic treatment regimens for surgical methods under the guidance of antibiotic use policies in different countries.

#### Length of stay and cost of hospitalization

A study conducted in China demonstrated that the length of hospital stay was significantly prolonged in patients with allergy labels (7.48 ± 6.11 days vs. 7.08 ± 6.57 days, *p* = 0.01). Similar findings have been reported in other countries and regions, where the presence of PALs may prolong hospital stays and increase healthcare costs (Kirven et al., 2021; Pérez-Encinas et al., 2022; Ali et al., 2024). A post-labeling reevaluation revealed a high incidence of mislabeling, with over 90% of patients with penicillin allergy labels successfully having the label removed (Goh et al., 2021). The penicillin provocation test in the outpatient department can effectively and safely remove the PAL, significantly reducing the burden of hospitalization. Similarly, Eric Macy reported saving $2,000 per patient annually in healthcare costs by detecting and removing the penicillin allergy label (Macy and Shu, 2017).

Our findings have significant implications for clinical practice, particularly in facilitating the removal of inappropriate penicillin allergy labels. By minimizing unnecessary penicillin allergy designations, patients can receive more precise medication, thereby avoiding the reliance on alternative therapies and reducing the waste of medical resources associated with mislabeling allergies. Furthermore, accurate allergy labeling enhances clinical outcomes, mitigates adverse reactions stemming from allergy misdiagnosis, and improves overall treatment efficacy. Future studies should further investigate the impact of penicillin allergy label removal on patient health, the duration of hospital stays, and the emergence of drug resistance. Additionally, research should concentrate on optimizing allergy assessment criteria and evaluating various allergy testing methodologies.

### Advantages and limitations

This meta-analysis integrated data from multiple independent studies to demonstrate an association between penicillin allergy labeling and serious adverse events, thereby providing more comprehensive evidence. This integration enhances the credibility of the conclusions and reflects the characteristics of a broader population. Various statistical analysis methods, including RR and 95%CI, were employed, alongside publication bias and sensitivity analyses, thereby enhancing the credibility and reliability of the results. The primary limitation of this analysis was that variations in population exposure may result in inaccurate outcomes. Upon reviewing the studies included in the analysis, we observed that all were clinical observations of hospitalized patients, whether infected or non-infected, and different studies may have encountered varying hospitalization events. Nonetheless, our comprehensive review revealed that the included articles were largely consistent in their trial design and analytical methods. The low heterogeneity among the studies was maintained, as confirmed by several of our analyses. Furthermore, the accuracy and stability of the results were significantly enhanced by excluding specific populations, such as kidney transplant recipients, from the mortality analysis. Although the study involved multiple centers, sample selection may have been biased, and certain populations might have been underrepresented, limiting the generalizability of the results. While meta-analyses can reveal correlations, they do not establish causation. Potential confounding factors, such as patients’ underlying health conditions and the use of other medications, may not have been fully considered in the analysis.

It is important to note that outpatient clinics are also a primary setting for the administration of penicillin, which may contribute to a range of adverse drug events. The analysis population primarily consisted of inpatients, and it was anticipated that future research would focus more on the role of outpatient clinics. Additionally, the included studies are predominantly from Western regions, and healthcare policies across countries influence clinical practices (e.g., in China, antibiotic skin tests may be repeated despite clear allergy labels), which may contribute to variations in results. It is expected that future studies conducted in diverse regions will provide additional clinical evidence.

The study ultimately emphasizes the importance of de-labeling and suggests the need for re-evaluation of non-allergic patients, which could promote improvements in clinical practice and the rational use of medical resources.

## Conclusion

Our findings indicated that penicillin allergy labels were associated with an increased risk of mortality in patients, alongside a higher likelihood of acute heart failure, greater ICU admission rates, and the need for mechanical ventilation. Interestingly, alternative drugs were, at times, more likely to induce adverse reactions than penicillin, especially in patients with pre-existing cardiovascular conditions. Consequently, we emphasized the importance of de-labeling and advocated for the re-evaluation of penicillin allergy labels. Such measures could have contributed to optimizing clinical practice and promoting the rational allocation of healthcare resources.

## Data Availability

The original contributions presented in the study are included in the article/supplementary material, further inquiries can be directed to the corresponding authors.
