# Supplementary material for: Association between penicillin allergy labels and serious adverse events in hospitalized patients: a systematic review and meta-analysis
Source: Front Pharmacol. 2025 Jan 6;15:1519522. doi: 10.3389/fphar.2024.1519522 (PMC11743256; doi:10.3389/fphar.2024.1519522)
Supplement: Supplementary file 2 [file DataSheet1.docx]

**Pubmed:**

(((penicillin allergy label[Title/Abstract]) OR (penicillin allergy[Title/Abstract])) OR (allergic to penicillin[Title/Abstract])) AND ((((hospitality[Title/Abstract]) OR (hospitality rate[Title/Abstract])) OR (hospitality length[Title/Abstract])) OR (hospitalization[Title/Abstract]))

#1 ((penicillin allergy label[Title/Abstract]) OR (penicillin allergy[Title/Abstract])) OR (allergic to penicillin[Title/Abstract])

#2 (((hospitality[Title/Abstract]) OR (hospitality rate[Title/Abstract])) OR (hospitality length[Title/Abstract])) OR (hospitalization[Title/Abstract])

Search: #1 AND #2

**Embase**

#1 'penicillin allergy label':ti,ab,kw OR 'penicillin allergy':ti,ab,kw OR 'allergic to penicillin':ti,ab,kw

#2 hospitalization:ti,ab,kw OR 'hospitality rate':ti,ab,kw OR hospitality:ti,ab,kw OR 'hospitality length':ti,ab,kw

Search: #1 AND #2

#3 'hospitalization' /exp

#4: #2 OR #3

#5: #1 AND #4

**WOS**

**#1 penicillin allergy (Topic) or penicillin allergy label (Topic) or allergic to penicillin (Topic)**

#2 hospitality (Topic) or hospitality rate (Topic) or hospitality length (Topic) or hospitalization (Topic)

Search: #1 AND #2

**Cochrane**

Search Name:

Date Run: 13/07/2024 20:19:50

Comment:

ID Search Hits

#1 (penicillin allergy label):ti,ab,kw OR (penicillin allergy):ti,ab,kw OR (allergic to penicillin):ti,ab,kw (Word variations have been searched) 386

#2 MeSH descriptor: [Hospitalization] explode all trees 20485

#3 (hospitalization):ti,ab,kw OR (hospitality):ti,ab,kw OR (hospitality rate):ti,ab,kw OR (hospitality length):ti,ab,kw OR (hospitalization rate):ti,ab,kw (Word variations have been searched) 251802

#4 #2 OR #3 254350

#5 #1 AND #4 107
